# Limited effect of intramuscular epinephrine on cardiovascular parameters during peanut-induced anaphylaxis: An observational cohort study

**DOI:** 10.1016/j.jaip.2020.08.041

**Published:** 2021-01

**Authors:** Paul J. Turner, Monica Ruiz-Garcia, Stephen R. Durham, Robert J. Boyle

**Affiliations:** a Section of Inflammation, Repair and Development, National Heart and Lung Institute, Imperial College London, London, United Kingdom; b Royal Brompton and Harefield Hospitals NHS Foundation Trust, London, United Kingdom

**Clinical Implications**

• Intramuscular injection with epinephrine had limited impact in reversing the decrease in stroke volume caused by peanut-induced anaphylaxis. These data question the effectiveness of intramuscular epinephrine alone to treat cardiovascular compromise during anaphylaxis and support the need for guidelines to incorporate effective adjuvant treatments in addition to intramuscular epinephrine in the management of refractory anaphylaxis.

Acute allergic reactions to peanut are associated with significant cardiovascular changes, with a reproducible decrease in stroke volume (SV), associated with increased peripheral blood flow.^1^ This may explain why changes in patient positioning during anaphylaxis can trigger decompensation, cardiorespiratory arrest, and death.^2^ In this observational study, we systematically evaluated changes in cardiovascular function after intramuscular (IM) epinephrine administration during peanut-induced anaphylaxis.

Peanut-allergic adults (18-45 years) with no known cardiovascular abnormality underwent repeated oral food challenge to peanut, as part of a clinical trial (TRACE Peanut study; ClinicalTrials.gov Identifier: NCT02665793). Detailed methods are described elsewhere.^1^ Participants were monitored continuously, using a Food and Drug Administration–approved monitor for noninvasive measurement of SV (Cheetah NICOM, Boston, MA). Heart rate (HR) monitoring was undertaken using a 12-lead Holter monitor (GE Healthcare, Chicago, IL). Patients were kept semirecumbent throughout to control for possible movement artifact. Written informed consent was obtained, and the study received ethics approval (NHS Human Research Authority, reference 15/LO/0286).

Fifty-seven adults were recruited, of whom 22 (39%) experienced anaphylaxis according to National Institute of Allergy and Infectious Disease/Food Allergy and Anaphylaxis Network criteria. Consistent with local practice, IM epinephrine (0.5 mg, injected with a 1 mL syringe and 21G needle into the mid-anterolateral thigh) was administered for all reactions presenting with objective respiratory and/or cardiovascular symptoms.^3^ Fourteen participants received at least 1 dose of IM epinephrine: 11 at the initial challenge and 3 (with non-anaphylaxis to the initial reaction) at the subsequent challenge. Baseline demographics are described in Table E1 (available in this article's Online Repository at www.jaci-inpractice.org). No participant had evidence of hemodynamic instability during reaction.

We observed a significant fall in SV (mean decrease: 9.7%, 95% confidence interval [CI]: 3.0 to 16.5; *P* = .0066) and an increase in HR (mean increase: 10 bpm, 95% CI: 5.0 to 16; *P* = .0011) and mean arterial blood pressure (MAP) (mean increase: 15.0%, 95% CI: 9.6 to 20.4; *P* < .0001) at the time of objective clinical reaction (OCR). There was no significant change in cardiac output (CO) at OCR (mean increase: 5.4%, 95% CI: −6.8 to 17.5; *P* = .36).

Administration of 0.5 mg IM epinephrine, although associated with improved symptoms, did not cause a consistent and clinically relevant increase in SV at any single timepoint (Figure 1). Using a composite measure (maximum change in the first 10 minutes after epinephrine), we observed a mean increase in SV of 9.4% (95% CI: 0.3 to 18.6; *P* = .029). HR also increased (mean: 11.9 bpm, 95% CI: 5.0 to 18.9; *P* = .0026), with an associated increase in CO (mean: 23%, 95% CI: 10 to 36; *P* = .0018) but not MAP (mean increase: 0.9%, 95% CI: −3.8 to 5.5; *P* = .70). Two participants received a second epinephrine dose; interestingly, in these subjects, epinephrine did not result in any further change in SV (Figure E1, available in this article's Online Repository at www.jaci-inpractice.org). Respiratory symptoms with an accompanying change in spirometry were present in 13 reactions. After epinephrine, there was a significant increase in forced expiratory volume in 1 second (mean: 14.1%, 95% CI: 1.3 to 26.9; *P* = .035) and peak expiratory flow rate (mean: 8.8%; 95% CI: 0.9 to 16.7; *P* = .033), although these patients also received nebulized salbutamol.

**Figure 1:**
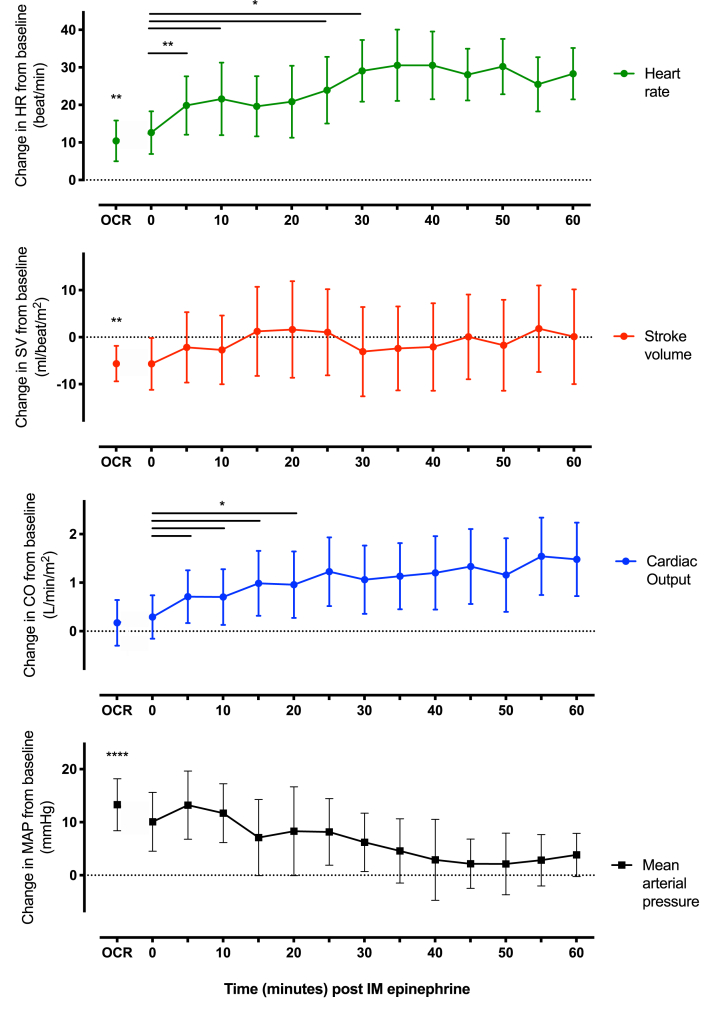
Changes in cardiovascular parameters at the time of objective clinical reaction (OCR) during peanut-induced anaphylaxis and after intramuscular (IM) epinephrine injection (at *T* = 0). Data are means with 95% CI, n = 14. ∗*P* < .05, ∗∗*P* < .01, ∗∗∗∗*P* < .0001, *t*-test. *CI*, Confidence interval; *CO*, cardiac output; *HR*, heart rate; *MAP*, mean arterial pressure; *SV*, stroke volume.

Eight subjects had anaphylaxis at a subsequent challenge but did not develop objective respiratory symptoms, and thus epinephrine was not administered. Anaphylaxis was associated with similar changes in HR, SV, and CO at OCR (Figure 2), irrespective of whether epinephrine was subsequently given. Comparing the effect of treatment (or not) with epinephrine in these subjects, epinephrine resulted in a significant increase in HR (compared with no epinephrine) in the first 10 minutes after injection (mean increase: 17 bpm, 95% CI: 4.1 to 29; *P* = .016) but no significant change in SV (mean increase: 11%, 95% CI: −10 to 32; *P* = .26) or CO (mean increase: 22%, 95% CI: −4.1 to 49; *P* = .086).

**Figure 2:**
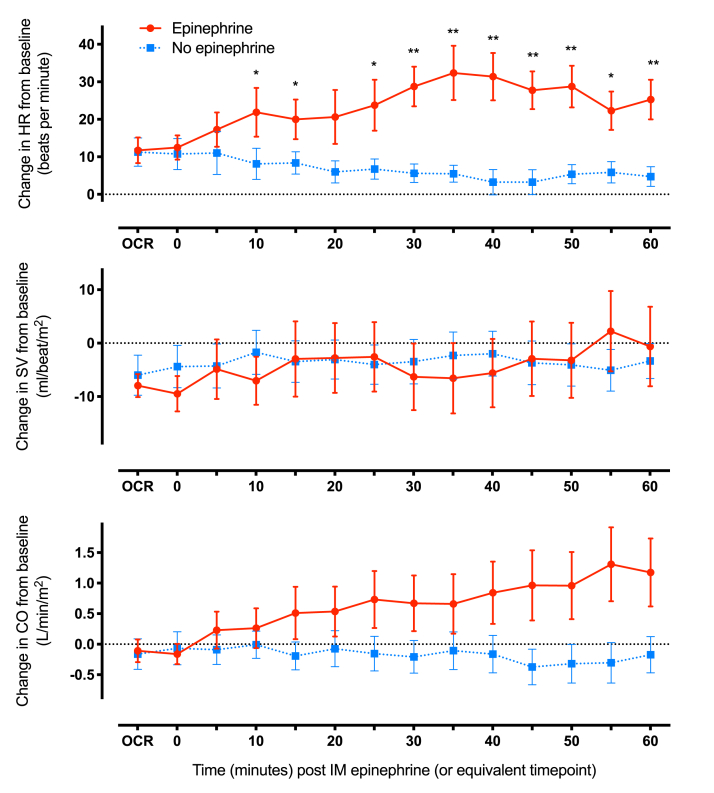
Changes in cardiovascular parameters at objective clinical reaction (OCR) with (in red) or without (in blue) IM epinephrine treatment (at *T* = 0), during peanut-induced anaphylaxis in participants who experienced anaphylaxis on more than 1 occasion, where only 1 reaction was treated with epinephrine. Data are means with 95% CI, n = 8. ∗*P* < .05, ∗∗*P* < .01, paired *t*-test. *CI*, Confidence interval; *CO*, cardiac output; *HR*, heart rate; *IM*, intramuscular; *SV*, stroke volume.

The evidence for IM epinephrine as first-line treatment for anaphylaxis is largely driven by retrospective case series and expert opinion.^4^ In this first in-human study of IM epinephrine treatment in subjects during food-induced anaphylaxis, epinephrine resulted in limited changes in HR and SV; any change in CO was essentially related to the increase in HR. In the analysis of patients who had anaphylaxis on more than 2 occasions, but only one was treated with epinephrine, a single dose of epinephrine did little to reverse the fall in SV induced by anaphylaxis, that is, there was little evidence of a positive *inotropic* effect of epinephrine. Although these data are limited by the relatively small sample size and nature of the reactions experienced (with no participant requiring more than 2 doses of IM epinephrine), they are consistent with a canine model of anaphylaxis in which IM epinephrine resulted in a negligible increase in SV.^5^

The majority of food-induced anaphylaxis reactions occurring in a community setting resolve without severe outcomes, whether or not they are treated with epinephrine.^6^ Although food-induced anaphylaxis is considered to be predominantly a respiratory event,^6^ cardiovascular changes are common, even in clinically mild reactions^1^; this might explain reports of fatal food anaphylaxis related to the postural change in young people without obvious severe symptoms.^2^ Severe reactions are often refractory to bolus epinephrine, but respond to a combination of low-dose intravenous epinephrine infusion in conjunction with fluid boluses.^7^ In this context, the limited impact of IM epinephrine on SV is concerning. Indeed, we reported a 5-fold greater sustained increase in SV after administration of intravenous fluids in the same series of patients, compared with that seen with IM epinephrine injection in this study.^1^

This does not negate the value of IM epinephrine in the management of anaphylaxis: we observed a clear beneficial impact on symptoms as well as lung function. Epinephrine causes vasoconstriction and reduces vascular leak, counteracting many of the adverse consequences of anaphylaxis. This is likely to explain why epinephrine administration in hemodynamically stable anaphylaxis patients is associated with a reduced risk of developing hypotension.^8^ Rather, our data emphasize the need for adjunctive treatments *in addition to* further IM epinephrine in those patients in whom initial epinephrine has only a limited effect. Further work is needed to identify determinants of a prompt physiological response to epinephrine during anaphylaxis. Around 10% of food-induced anaphylaxis reactions fail to respond to a single dose of IM epinephrine.^9^ Current guidelines tend not to recommend intravenous fluids in the absence of hemodynamic instability,^3^^,^^4^ yet a lack of response to initial epinephrine may be due to insufficient drug delivery secondary to reduced venous return.^1^ We therefore advocate for rapid escalation with early intravenous fluid therapy where anaphylaxis is refractory to initial IM epinephrine, even in patients without obvious hemodynamic instability.

## Figures and Tables

**Figure E1:**
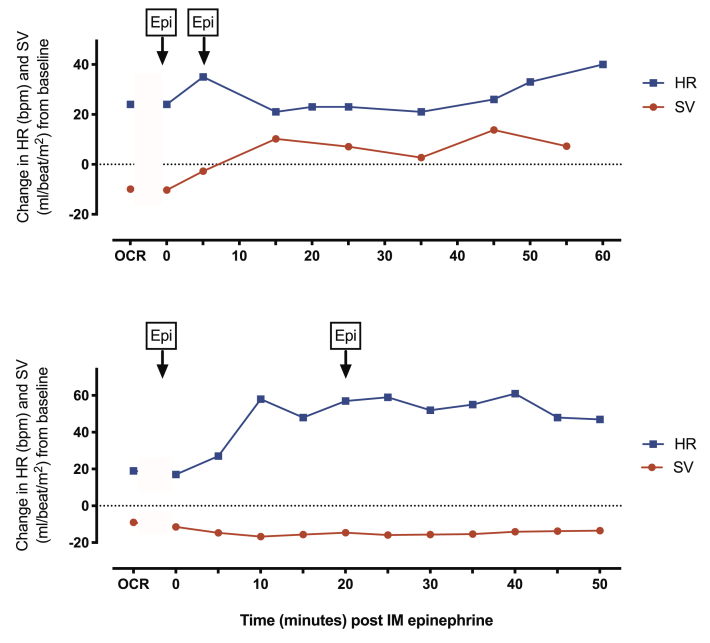
Change in heart rate (HR) and stroke volume (SV) in 2 participants who received 2 doses of IM epinephrine. *IM*, Intramuscular; *OCR*, objective clinical reaction.

**Table E1:** Characteristics of the study population

Characteristic	Overall cohort (N = 57)	IM epinephrine given (N = 14)
Age at enrollment (y)	24 (20, 29)	23 (19, 27)
Sex (female)	30 (53%)	7 (50%)
Asthma	29 (51%)	9 (64%)
Rhinitis	40 (70%)	10 (71%)
Eczema	27 (47%)	6 (43%)
Total IgE (kUA/L)	221 (107, 576)	153 (87, 234)
Specific IgE to peanut (kUA/L)	10.2 (3.3, 31.9)	11.4 (3.6, 25.6)
Specific IgE to rAra h 2 (kUA/L)	7.4 (2.0, 20.4)	6.6 (2.1, 14.9)
SPT wheal size to commercial peanut extract	11 mm (9, 15)	13 mm (9, 19)
Cumulative reaction dose (mg peanut protein)	133 (33, 433)	183 (33, 683)
Baseline mast cell tryptase (ng/mL)	2.7 (2.2, 3.7)	2.6 (2.1, 3.2)

Continuous variables are described as median (IQR) for nonparametric data.

*IM*, Intramuscular; *IQR*, interquartile range; *SPT*, skin prick test.
